# Cystic Fibrosis Transmembrane Conductance Regulator Folding Mutations Reveal Differences in Corrector Efficacy Linked to Increases in Immature Cystic Fibrosis Transmembrane Conductance Regulator Expression

**DOI:** 10.3389/fphys.2021.695767

**Published:** 2021-10-26

**Authors:** Kathryn W. Peters, Xiaoyan Gong, Raymond A. Frizzell

**Affiliations:** Department of Pediatrics, University of Pittsburgh School of Medicine, Pittsburgh, PA, United States

**Keywords:** cystic fibrosis, CFTR, mutations, corrector efficacy, SUMOylation

## Abstract

**Background:** Most cystic fibrosis is caused by mutations in the cystic fibrosis transmembrane conductance regulator (CFTR) gene that lead to protein misfolding and degradation by the ubiquitin–proteasome system. Previous studies demonstrated that PIAS4 facilitates the modification of wild-type (WT) and F508del CFTR by small ubiquitin-like modifier (SUMO)-1, enhancing CFTR biogenesis by slowing immature CFTR degradation and producing increased immature CFTR band B.

**Methods:** We evaluated two correction strategies using misfolding mutants, including the common variant, F508del. We examined the effects on mutant expression of co-expression with PIAS4 (E3 SUMO ligase), and/or the corrector, C18. To study the impact of these correction conditions, we transfected CFBE410- cells, a bronchial epithelial cell line, with a CFTR mutant plus: (1) empty vector, (2) empty vector plus overnight 5 μM C18, (3) PIAS4, and (4) PIAS4 plus C18. We assessed expression at steady state by immunoblot of CFTR band B, and if present, band C, and the corresponding C:B band ratio. The large PIAS4-induced increase in band B expression allowed us to ask whether C18 could act on the now abundant immature protein to enhance correction above the control level, as reported by the C:B ratio.

**Results:** The data fell into three mutant CFTR categories as follows: (1) intransigent: no observable band C under any condition (i.e., C:B = 0); (2) throughput responsive: a C:B ratio less than control, but suggesting that the increased band C resulted from PIAS4-induced increases in band B production; and (3) folding responsive: a C:B ratio greater than control, reflecting C18-induced folding greater than that expected from increased throughput due to the PIAS4-induced band B level.

**Conclusion:** These results suggest that the immature forms of CFTR folding intermediates occupy different loci within the energetic/kinetic folding landscape of CFTR. The evaluation of their properties could assist in the development of correctors that can target the more difficult-to-fold mutant conformations that occupy different sites within the CFTR folding pathway.

## Introduction

The cystic fibrosis transmembrane conductance regulator (CFTR) folding scheme is complex. It is managed by a set of chaperones, trafficking, and degradation components, a system collectively termed as “proteostasis” (Balch et al., 2011; Amaral and Balch, 2015). These components dictate the ability of CFTR to fold and traverse the endoplasmic reticulum quality control (ERQC) checkpoints that determine its transport from the endoplasmic reticulum (ER) to the apical plasma membrane (PM) of epithelial cells where it functions as a cAMP/PKA-regulated anion channel (Cliff and Frizzell, 1990). These ERQC checkpoints are incompletely understood for complex proteins such as CFTR. Previously, we discovered physical and functional modifications of CFTR with the small ubiquitin-like modifier (SUMO; Ahner et al., 2013). We initially found that the common mutant, F508del CFTR, was targeted for degradation by the small heat shock protein, Hsp27, which binds to the SUMO E2 enzyme, Ubc9 (Ahner et al., 2013). This interaction leads to the modification of the F508del mutant with the nearly identical SUMO-2 and SUMO-3 paralogs, which are capable of forming SUMO-2/3 poly-chains. These SUMO poly-chains are recognized by the ubiquitin ligase, RNF4, leading to the proteasomal degradation of the mutant (Gong et al., 2019).

Conversely, protein interaction arrays identified the SUMO E3 enzyme, i.e., the Protein Inhibitor of Activated STAT isoform 4 (PIAS4), whose overexpression increased both wild-type (WT) and F508del CFTR biogenesis in airway epithelial cells. For WT CFTR, PIAS4 increased immature CFTR 3-fold and mature CFTR 2-fold (Gong et al., 2019). In subsequent studies, PIAS4 also slowed immature F508del degradation 3-fold and stabilized mature CFTR at the PM. PIAS4 modified F508del CFTR with the SUMO-1 paralog, which does not form poly-chains, reduced its conjugation to SUMO-2/3 and ubiquitin, both *in vitro* and *in vivo*, and blocked the action of RNF4 on mutant CFTR degradation. These findings indicated that a SUMO paralog switch during WT or mutant CFTR biogenesis can direct the protein to different outcomes, i.e., biogenesis vs. degradation.

A primary effect of PIAS4 on CFTR is to increase the expression of immature CFTR. For F508del, this generates a lesser amount of mature protein and increases the impact of correctors on the production of mature F508del band C, which was functional at the PM based on the cell surface expression of F508del fluorogen-activated protein (FAP; Holleran et al., 2012, 2013) and the measurements of patch-clamp current (Gong et al., 2019). The aim of this study was to determine if the SUMO-1-related impact on CFTR biogenesis extends to rarer CFTR folding mutations, as enumerated in the CFTR2 database^1^. In view of its potential therapeutic importance, we focused predominantly on the actions of PIAS4 on the biogenesis of missense mutants in different CFTR domains.

## Materials and Methods

### Cell Culture

The CFBE41o- cells, a bronchial epithelial cell line, were developed by DC Gruenert (Kunzelmann et al., 1993), kindly provided by JP Clancey (University of Alabama, Birmingham), and used for transient transfections of WT CFTR, F508del, and CFTR2 mutants. Cells were maintained at 37°C in a humidified chamber with 5% CO_2_ and were cultured in complete media as follows: Earle’s Minimum Essential Medium, supplemented with 10% fetal bovine serum (HyClone, Logan, UT), 50 U/ml penicillin/50 μg/ml streptomycin, and 2 mM glutamine (Gibco, Waltham, MA). This formulation (lines 196–200) without antibiotics was followed for preparing semi-starvation media. CFBE-F508del cells for limited trypsin proteolysis were kindly provided by WE Balch (Scripps Research, La Jolla, CA, United States) and grown as above except 2 μg/ml puromycin (InvivoGen, San Diego, CA) was included in complete media as a selection agent.

### Plasmids

To study the effects of PIAS4 on CFTR expression, we examined transiently transfected WT CFTR, F508del, and 17 CFTR2 mutants, which included P67L, G85E, E92K, R117H, R334W, R347P, A455E, S492F, V520F, S549R, R560K, R560T, D614G, L1065P, R1066C, L1077P, M1101K, and N1303K. These constructs were the generous gift of PJ Thomas and Linda Millen, UTSW. Sequences were inserted into the Not1 site of the mammalian expression vector pBI-CMV2 (Clontech, Mountain View, CA; Vetter et al., 2016).

### Transfection of CFBE41o- Cells

The CFBE41o- cells were propagated in flasks until about 90% confluent and then seeded onto six-well cell culture plates (Corning Costar, Tewksbury, MA) so that cells would be approximately 70% confluent the next day. Several hours before transfections, complete media was replaced with semi-starvation media. Transient transfections were accomplished using a 1:3 ratio of μg DNA to μl Lipofectamine 2000 (Invitrogen, Carlsbad, CA) following the instructions of the manufacturer. We utilized four experimental groups with each CFTR2 mutant as follows: (1) empty vector, (2) empty vector plus C18 (aka VRT-534; Eckford et al., 2014), (3) PIAS4, and (4) PIAS4 plus C18. Culture plates of transfected cells were placed in the incubator and remained there overnight. Of note, 24 h later, semi-starvation media was replaced with complete media. Corrector was added to the appropriate wells and allowed to incubate for 18 h.

### Sodium Dodecyl Sulfate-Polyacrylamide Gel Electrophoresis (SDS-PAGE) and Transfer

On Day 2 after transfection, all cells were rinsed with phosphate-buffered saline (PBS) and then lysed [20 mM *Tris* base, 150 mM NaCl, 10% glycerol, 1% Triton X-100, 2 mM ethylenediaminetetraacetic acid (EDTA), pH 8.0; one protease inhibitor cocktail tablet (Roche: 46264500) was added to 10 ml lysis buffer], and protein concentrations were determined by a bicinchoninic acid (BCA) assay (Pierce, Rockford, IL), according to the instructions of the manufacturer. We employed BCA assays in duplicate, averaged for each sample so that equal amounts of protein were loaded for immunoblots. We chose to use each mutant and empty vector as a baseline, permitting the comparison of corrector-treated samples.

Laemmli buffer (2×) was added to equal amounts of lysates that were heated for 10 min at 40°C in a dry bath incubator, loaded onto 7% polyacrylamide mini gels, and electrophoresed at 100 V constant (BioRad PowerPac 3000, Hercules, CA) until the dye front ran off the gel. Gels were transferred to 0.025 M *Tris*/0.192 M glycine buffer (National Diagnostics, Atlanta, GA) containing 20% MeOH onto whetted Immobilon-FL (Millipore, St. Louis, MO) polyvinylidene fluoride (PVDF) membranes, constantly at 38 V overnight.

### Isolation of Microsomes From CFBE-F508del Cells

The CFBE-F508del cells were grown in flasks and when nearly 90% confluent, seeded onto 100 mm, treated (i.e., vacuum gas plasma) tissue culture dishes (Corning Falcon, Tewksbury, MA) so that cells would be approximately 70% confluent the following day. Complete media was removed, and semi-starvation media was added. Transfections were performed with 1 μg DNA to 2 μl Lipofectamine 2000 (Invitrogen) using an empty vector for controls or PIAS4 to observe its effects on CFTR such as the formation of proteolytic fragments. On Day 1, incomplete media was exchanged for complete. Two days after transfection, cells were washed and scraped in Dulbecco’s PBS (DPBS) and then forced gently 15 times through a cell disrupter using a ball with a diameter of 0.1558 inches (Industrial Tectonics Inc., Dexter, MI). The nuclear and mitochondrial pellet was removed by centrifugation at 4,000×*g* for 10 min, and the supernatant was harvested and spun at 100,000×*g* for 60 min. The protein concentration of the resulting microsomal pellet was determined by the BCA assay.

### Limited Proteolysis and Cycloheximide Chase

Of note, 30 μg of microsomal vesicles from CFBE-F508del cells were placed into each of the five tubes. Due to the five different concentrations of TPCK-treated trypsin (0, 20, 40, 80, or 240 μg/ml) that would be used from the 1 mg/ml stock, various amounts of DPBS were included so that all tubes would have equal final volumes. To the vesicles in DPBS, the addition of trypsin to each tube was carefully timed and staggered so that each tube undergoing proteolysis was on ice exactly for 15 min. Precisely at the 15-min mark, STOP solution (i.e., 8 mg soybean trypsin inhibitor, 10 μg leupeptin, 10 μg pepstatin, and 0.8 μl phenylmethylsulfonyl fluoride) was added (again staggered). Finally, 6 × Laemmli sample buffer was added, and lysates were heated at 40°C for 10 min. Equal amounts of the extracts were loaded onto 7%/12% step polyacrylamide gels, then electrophoresed, and transferred as mentioned earlier. Cycloheximide chase was performed as described in the study by Gong et al. (2019).

### 7%/12% Step Gels

The assembled mini gel plates were positioned in a casting stand, and three marks at 2.5, 5, and 6 cm from the bottom of the short plate were placed for identification where layers would begin and/or end. The 12% polyacrylamide solution was pipetted to the first mark at 2.5 cm and covered with saturated butanol which–when the gel was polymerized–was removed. Then, 7% polyacrylamide was pipetted to the second mark at 5 cm and covered with saturated butanol. Finally, the stacking gel solution was added to the 6-cm mark, a comb was inserted, and the gel was placed in a mini-Protean II Electrophoresis Cell after polymerization. Gels were run and transferred as mentioned earlier.

### Immunoblotting

After transfer, membranes were rinsed with PBS and then blocked in Odyssey Blocking Buffer (Li-Cor #927-40000, Lincoln, NE, United States) (1:2 blocking buffer:PBS) for 1 h. Membranes were then (1) incubated with monoclonal antibody (1:2 blocking buffer:PBS, 0.1% Tween 20) for 1 h, (2) rinsed three times in PBS/0.1% Tween, (3) incubated in 2° antibody with an attached fluorophore (Li-Cor IRDye 800 CW Donkey anti-Mouse IgG; in 1:2 blocking buffer:PBS, 0.1% Tween 20, 0.01% SDS) for 1 h, and finally (4) rinsed three times in PBS/0.1% Tween. The membrane was dried and viewed on an Odyssey Classic IR Imaging System (Li-Cor, Lincoln, NE, United States).

### Antibodies

Mouse monoclonal antibody, 596 (University of North Carolina, Chapel Hill) (epitope amino acids 1,204–1,211 of the second nucleotide-binding domain of CFTR), was used at a dilution of 1:10,000 for the analyses of transient transfections. Monoclonal 13-4 (Millipore), which recognizes an epitope at the N-terminus of CFTR, amino acids 24–35, was used at 1:100 to analyze the 40 kDa proteolytic fragments that consist of N-terminus to just beyond MSD1 of CFTR (Cui et al., 2007).

## Results

**FIGURE 1:**
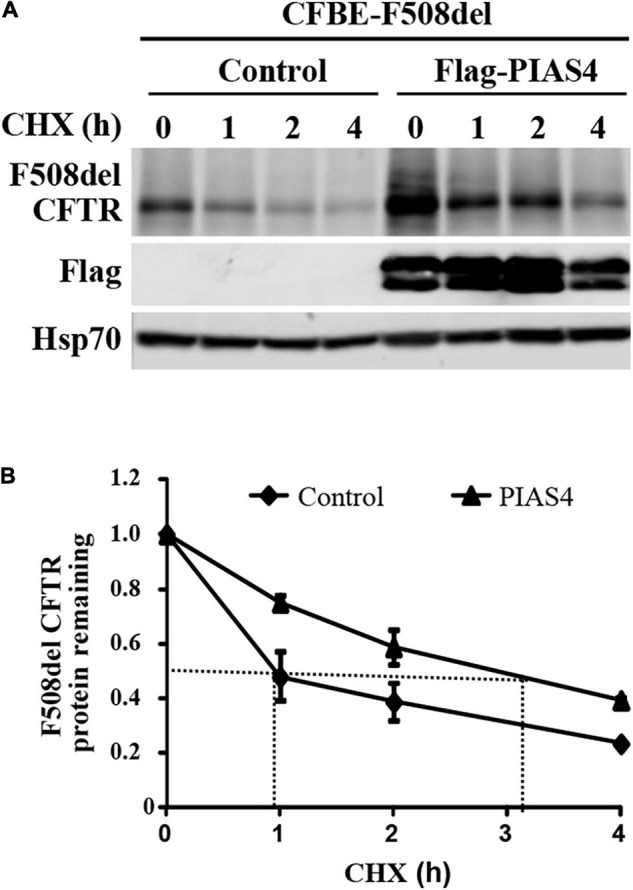
PIAS4 slows F508del cystic fibrosis transmembrane conductance regulator (CFTR) degradation. **(A)** PIAS4 overexpression increased the expression of F508del CFTR in CFBE airway cells; its impact was greatest on immature CFTR, band B (Gong et al., 2019). This result was clarified by cycloheximide chase experiments, which showed that CFBE cells overexpressing PIAS4 exhibited a 3-fold decrease in the rate of F508del CFTR degradation. **(B)** Following immunoblot, blot densities from three independent experiments were averaged to construct the relations for F508del CFTR (band B) remaining as a function of time, with values from each experiment normalized to the mean densities at the beginning of the CHX chase (*t* = 0). PIAS4 itself may undergo posttranslational modification, which likely explains the pattern of FLAG epitope staining.

In cystic fibrosis bronchial epithelial (CFBE) airway cells, where all experiments were performed, our prior study demonstrated that PIAS4 overexpression increased the expression of both WT and F508del CFTR; for both proteins, its impact was greatest on the immature form, band B CFTR. In this study, this result was supported by cycloheximide chase experiments (Figure 1), which showed that CFBE airway cells overexpressing PIAS4 exhibited a 3-fold decrease in the rate of degradation of F508del CFTR. This is shown by differences in the time required for F508del to reach 50% decay in its rate of degradation, as indicated by the dashed lines in Figure 1.

**FIGURE 2:**
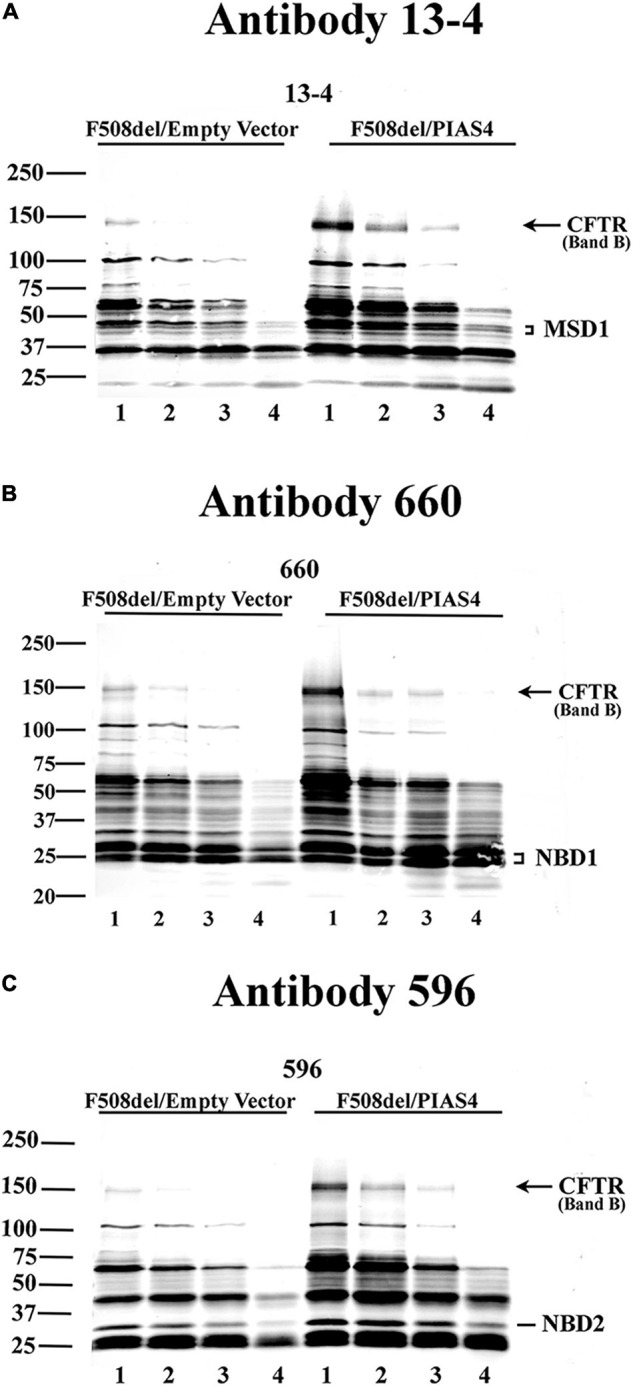
The impact of PIAS4 on the protease sensitivity of F508del CFTR. Microsomal vesicles were isolated from transfected CFBE-F508del cells and subjected to cellular disruption as described in the “Materials and Methods” section. The microsomal pellet was subjected to 20, 40, 80, or 240 μg/ml TPCK trypsin, lanes 1–4, respectively, for 15 min each, resolved on 7%/12% step gels and transferred. **(A)** Proteins were blotted with CFTR monoclonal 13-4 targeting the CFTR N-terminus. PIAS4 augmented band B expression and protected CFTR tryptic fragments generated by trypsin-mediated hydrolysis. Quantitation of the 13-4 gel yielded relative density values: at MW bands of 50–75 kDa, for control: 1.0, 0.54, 0.34, and 0.056; and for PIAS4 treated: 1.0, 0.80, 0.40, and 0.11. Density at MW bands ∼40–50 kDa for control: 1.0, 0.56, 0.40, and 0.16; and for PIAS4 treated: 1.0, 0.75, 0.46, and 0.29. **(B,C)** Other anti-CFTR monoclonals gave qualitatively similar results that identified fragment patterns for NBD1 (Ab #660) or NBD2 (Ab #596), respectively, which also provided evidence of protease protection. Thus, the densities of identifiable domains of F508del CFTR were increased for all fragments, as PIAS4 stabilized domain fragments against protease activity.

In addition, any changes in the structure of F508del CFTR due to PIAS4 action/modification were assessed using the protease protection analysis. This approach uses expressed CFTR to detect partially unfolded structures that emerge during graded protease cleavage of the protein. Sites in a folded, compact protein are less accessible, i.e., they are protected from protease cleavage, as opposed to proteins in a more extended, partially denatured conformation, which displays different cleavage patterns. We detected the action of PIAS4 on the protease sensitivity of F508del CFTR using microsomal vesicles isolated from transfected CFBE-F508del cells by the addition of graded protease as described in the “Materials and Methods” section. Following digestion, the microsomal pellets were transferred and blotted with CFTR monoclonal antibodies 13-4, 660, and 596 (Cui et al., 2007). Figure 2 shows the results of tryptic fragment detection by these site-specific monoclonals as follows: N-terminus targeted Mab 13-4, NBD1 targeted Mab 660, and NBD2 targeted Mab 596; these blots indicated that the density of band B CFTR, as well as fragments from the specific domains which these antibodies target, as shown in the figures, were detected under both control conditions and were increased during the expression of PIAS4; band B is indicated in all blots and was significantly increased by the expression of PIAS4 relative to control as proteolysis was progressed. Antibody 13-4 protected MSD1 as protease concentration was increased; NBD1 was protected by Mab 660 and was more prevalent with the expression of PIAS4; similarly, MND2 was protected from trypsin by Mab 596. Thus, the primary result obtained with all antibodies was the increased detection of tryptic fragments of F508del CFTR in cells expressing PIAS4, but their mobilities were similar with and without the expression of SUMO E3. This suggests that the domain conformations of this multisubunit protein were not influenced by the posttranslational modification by SUMO during the augmentation of immature CFTR expression.

To begin the mutant analyses, we chose the following 18 disease-causing missense mutations from the CFTR2 database (see text footnote 1), namely, P67L, G85E, E92K, R117H, R334W, R347P, A455E, S492F, V520F, R560K, R560T, S549R, D614G, L1065P, R1066C, L1077P, M1101K, and N1303K. Generally, these variants encode misfolded CFTR proteins, although they may additionally tender proteins of varying competency in either structure or function. F508del was included as a reference variant due to its prevalence in the disease literature. Other than F508del, these mutations account for CF disease in patients ranging from 2.5 to 0.01% worldwide. The impact of the disease of these mutations is summarized in Supplementary Table 1, which provides sweat chloride in mEq/L and also the percentage of patients with that mutation reported in the CFTR2 database who are pancreatic insufficient. These disease parameters show a direct relationship, as anticipated.

We examined the effects of PIAS4, a SUMO E3 ligase, on the expression of the CFTR2 mutants listed earlier and in the Supplementary Table 1. In general, SUMO E3s mediate the selection of target protein for posttranslational SUMO modification, they catalytically enhance the kinetics of SUMO modification, and they can also determine the SUMO paralog used for conjugation (Gareau and Lima, 2010; Liebelt and Vertegaal, 2016). PIAS4 is expressed in human bronchial epithelial cells, and it mediates the SUMOylation of CFTR by SUMO-1, reduces its modification by SUMO-2/3, and decreases its ubiquitylation, thereby reducing its degradation (Gong et al., 2019).

### Rationale

**FIGURE 3:**
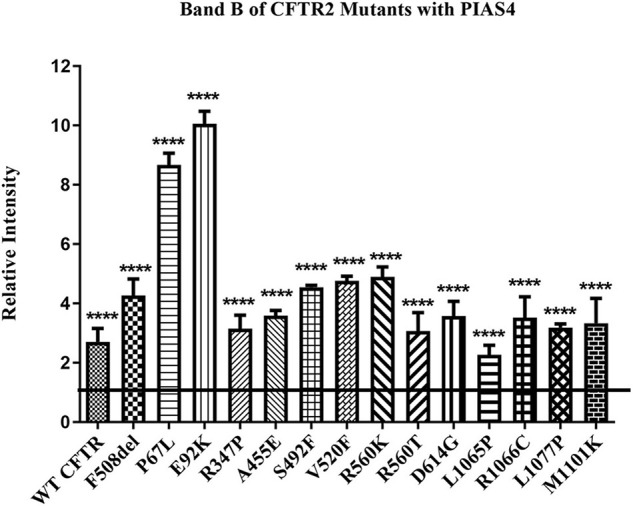
Effect of PIAS4 on band B expression in CFTR2 mutants. CFBE41o- cells were transfected with a CFTR2 mutant with or without PIAS4. SDS–PAGE was performed, and intensities of the bands were analyzed on a Li-Cor Odyssey IR imaging system. The control band B level for each mutant was set to a value of 1.0, depicted by the horizontal line, whereas the height of each bar represents band B level relative to that of the control. PIAS4 co-expression increased band B intensity for each mutant. ****P < 0.0001.

We took advantage of these effects of PIAS4 to ask whether increasing the amount of expressed immature mutant CFTR would enhance the efficacy of a CFTR corrector. First, we asked whether a significant increase in band B expression was observed for every CFTR2 mutant examined when co-expressed with PIAS4. Many of these missense mutations formed less or no mature CFTR when expressed individually in CFBE41o- airway cells; however, as shown in Figure 3, all of these variants expressed significantly more immature CFTR in response to PIAS4 overexpression. To evaluate their responses to PIAS4 and corrector, the data were obtained from parental CFBE41o- airway cells 2 days after transfection with a CFTR variant, with or without PIAS4. Total DNA and transfection reagent were maintained constant by empty vector addition.

**FIGURE 4:**
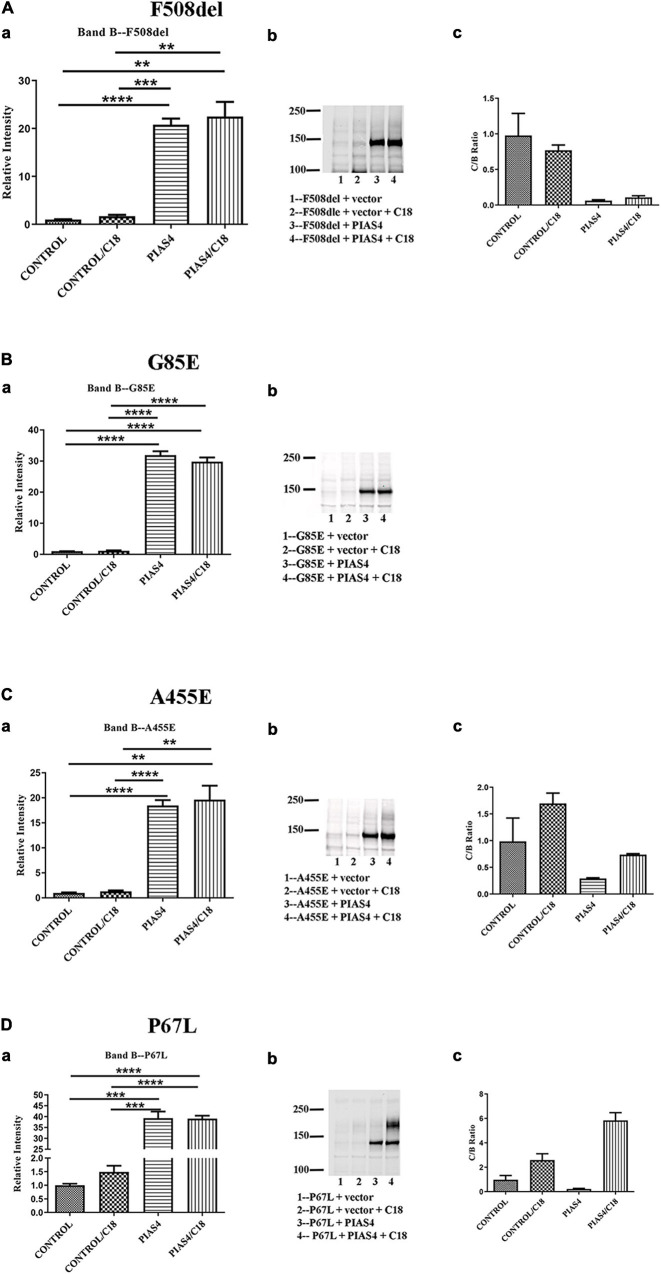
Representative data illustrating the analysis for each of the three categories. **(A)** Throughput responsive mutant F508del, which is presented as a benchmark for the analysis in view of its significance in CF. **(B)** Intransigent mutant G85E. The intensity data from three blots generated the mean data for the bar graph. No band C was detected. **(C)** Throughput responsive mutant A455E. The intensities of the three blots show bands B and C produced by the co-expression of PIAS4 with/without C18. The C:B ratio shows that although band C is produced, the amount is less than control. **(D)** Folding responsive mutant P67L. The representative blots show the significant generation of band C with the co-expression of PIAS4 and in response to C18. The C:B ratio was 6-fold higher than the control value, indicating corrector-induced mature CFTR generation exceeding the PIAS4-enhanced band B level. *P = 0.01–0.05; **P = 0.001–0.01; ***P = 0.0001–0.001; ****P = 0.0001.

Prior to cell lysis, control and PIAS4-treated cells were incubated overnight with corrector C18, a precursor of VX-809 (Lumacaftor) generated by medicinal chemistry and supported by the Cystic Fibrosis Foundation (Van Goor et al., 2011). These studies were initiated using C18 aka VRT-534 at a time when VX-809 was still not available. C18 and VX-809 are in the same chemical family, and they do not possess additive effect, implying that they share the same mechanism of action (MOA). Figure 4 shows the examples of the data analysis for a few representative mutations. Densities were quantified using a Li-Cor Odyssey IR Imager. Although some of the control CFTR bands (i.e., except PIAS4 or C18) are difficult to discern due to the large subsequent effects of PIAS4 on band B expression, the imaging sensitivity was subsequently adjusted so that bands in all lanes were readily visible before they were quantified. For example, the F508del data in Figure 4A were recorded at a higher sensitivity to reveal its control bands. The Odyssey imager has six logs of linear range.

Some variants, e.g., G85E [i.e., the introduction of a charged side chain into transmembrane domain 1 (TM1)], are incapable of generating mature CFTR in response to corrector despite a large PIAS4-induced increase in immature G85E CFTR (Figure 4B). We labeled this class of mutants as “Intransigent for their inability to respond to the PIAS4-induced increase in immature CFTR.” Other mutants, such as A455E, responded to the induction of PIAS4 of band B with visible but relatively small increases in band C; however, as observed for F508del, the percentage of increase with corrector plus PIAS4 did not rise above the level observed with corrector alone (Figure 4C). Therefore, we referred to mutants in this category as “throughput responsive.” These mutants appear to increase band C production due to the PIAS4-generated increase in band B. Finally, we found a larger group of variants in which the combined effects of PIAS4 plus corrector on band C expression exceeded the relative effect of corrector alone, as can be noted in the case of P67L (Figure 4D). These were termed as “folding responsive” mutants.

**FIGURE 5:**
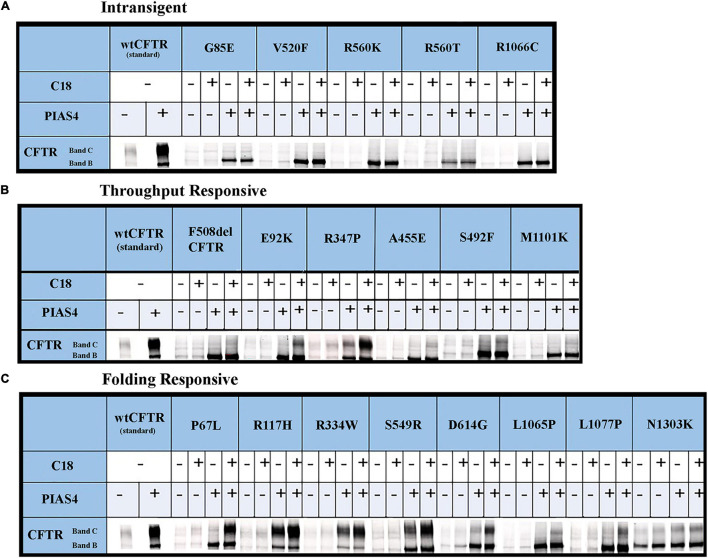
Immunoblots illustrating CFTR folding variants: the raw data. CFTR folding mutations that produce different levels of band C under control conditions can be distinguished by their responses to corrector C18 as PIAS4 elicits CFTR band B overexpression. See the text in the “Results” section and Figure 4 legend for the identification of mutants and the definition of their categories. The corrector C18 was identified during medicinal chemistry that led to the discovery of corrector VX-809/Lumakaftor (Van Goor et al., 2011).

**FIGURE 6:**
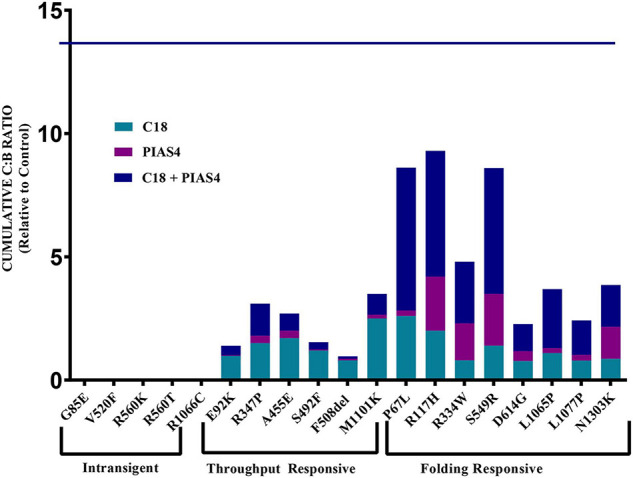
Cumulative C:B ratio for all groups. The C:B ratio for each treatment, relative to control, shows patterns of responses in each of the identified groups. The Intransigent group showed no band C production. The throughput responsive group shows some band C production, but the C:B ratio for C18 + PIAS4 was less than that due to C18 alone. In the folding responsive group, the C:B ratio for PIAS4 + C18 was greater than that due to C18 alone, indicating that C18 produced folding beyond that expected from the PIAS4-induced production of band B. The horizontal line at 13.8 on the ordinate represents the C:B ratio of wild-type CFTR for comparison.

The data for each of the categories discussed in this study are provided in Figure 5 and can be categorized by quantifying the intensity of band B under control conditions, under control plus C18, under co-expression with PIAS4, or with PIAS4 plus C18 treatment. Representative blots are provided for each of the mutants examined and the categories of response to which they belong. Finally, in each representation of these categories, the C:B ratios under these conditions were determined, except for the Intransigent group in which there was no detectable band C production. The cumulative C:B ratios for all mutants and all groups are shown in Figure 6. The “Discussion” section will cover the literature observations on the mutants presented in Figure 6 and the manner in which they conform to these categories.

**FIGURE 7:**
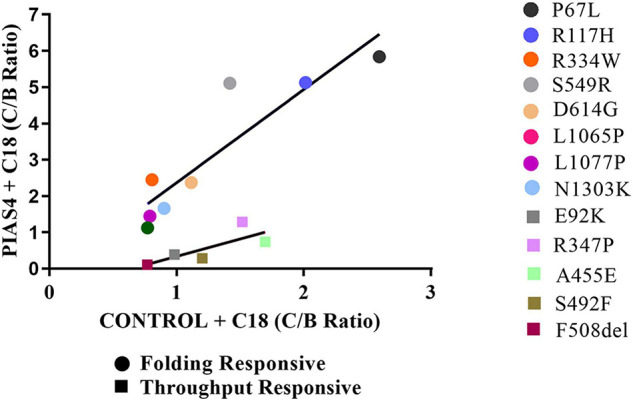
Graphical representation of the groups. C:B ratios for CFTR2 mutants in the folding responsive (circles) and throughput responsive (squares) groups relating values for PIAS4 + C18 to values for C18 alone. The slope of this relation for the throughput responsive mutants was 0.96 and that for the folding responsive mutants was 2.8. Folding responsive: *y* = 2.6*x* – 0.22; *R*^2^ = 0.76. Throughput responsive: *y* = 0.96*x* – 0.62; *R*^2^ = 0.61.

Another way to quantify the properties of these groups is illustrated graphically in Figure 7. Since we are plotting PIAS4 + C18 on the ordinate against Control + C18 on the abscissa, the slopes of the lines describing these mutants provide the increase (i.e., amplification) of mature CFTR expression enabled by the PIAS4-induced increase in immature CFTR. It was clear from this graphical representation that the amplification of mature CFTR expression varied in specific variants and with the mutation group with which they were associated. The slope of the throughput responsive data was 0.96, consistent with the concept that increased band C with corrector was driven primarily by increased immature CFTR production due to the expression of the SUMO E3. For the folding responsive group, the slope was 2.6, indicating that almost three times more the band C was generated by the corrector for this set of variants, and reflecting the ability of the corrector to take advantage of, or amplify, the PIAS4-induced increase in immature CFTR. These findings support the actions of C18 and PIAS4 and the relation between CFTR mutant sensitivities to the actions of a CFTR corrector as an amplifier.

## Discussion

We examined the effects of PIAS4, a SUMO E3 ligase, on the ability of CFTR variants to mature in response to corrector C18 when the immature CFTR level was elevated. This SUMO E3 appears to facilitate the targeting of CFTR in the cytoplasm of airway cells and employs the SUMO-1 paralog in its posttranslational modification of CFTR protein. PIAS4 assists in the stabilization of nascent CFTR (Figure 1), protects the protein from proteolysis (Figure 2), and blocks the modification of CFTR by SUMO-2/3 and thereby decreases its ubiquitylation by the RNF4 ubiquitin ligase (Gong et al., 2019). By reducing the rate of CFTR degradation, PIAS4 leads to substantial increases in the steady-state expression of immature F508del CFTR (Figure 3), which we attempted to use in a mutation agnostic manner to evaluate corrector impact. PIAS4 also produced a significant increase in the expression of WT CFTR band B and band C, suggesting that this SUMO E3 was able to access the physiological pathways for CFTR production, which are present in the airway cell line employed in this analysis (Gong et al., 2019).

We took advantage of these actions of PIAS4 to ask whether increasing the expression of immature mutant CFTR would enhance the efficacy of correctors in a general manner, a property that has been attributed to small molecules termed as “CFTR amplifiers” (Giuliano et al., 2018). In this study, every rare CFTR2 mutant examined showed increased steady-state band B expression when co-expressed with PIAS4, although the extent of the response varied with mutation (Figure 3). The impact of C18 also varied among the different CFTR2 folding variants. As noted earlier, C18 was identified from medicinal chemistry campaigns that led to the discovery of the first clinically evaluated corrector, VX-809^2^. C18 and VX-809 have similar correction efficacy, and their effects on CFTR maturation are non-additive (Hodos et al., 2020), suggesting a shared MOA.

When the correction data were compared and displayed graphically, the mutants fell into separate, identifiable groups (Figures 4–7). For each mutant, we plotted the relative biochemical response to C18 (C:B ratio) under otherwise control conditions on the abscissa and the response to C18 plus PIAS4 (also C:B) on the ordinate. Thus, for each mutant, its position on the graph (Figure 7) was determined by its response to C18 in the *x*-dimension, and its response to C18, after the expression of PIAS4 to elevate immature CFTR, in the *y*-dimension, i.e., in other words, the extent of amplification of the impact of the corrector. Using this approach, the mutant data fell into three groups. In the following sections, we covered the properties of each group and provided the selected literature citations and the recognized properties of selected mutants.

Regarding CFTR correctors, the common F508del mutant was relatively insensitive to early correctors such as the structurally similar C18 and VX-809 (Holleran et al., 2012, 2013; Eckford et al., 2014; Chung et al., 2016; Liu et al., 2018). Thus, the relatively low position of the common mutant on the corrector efficacy plots (Figure 7) should not be surprising; both C18 and VX-809 have been categorized as type I F508del CFTR correctors, which are considered to target NBD1/membrane-spanning domain interfaces. These first-generation correctors have been followed by other compounds with improved efficacy due to their ability to target different CFTR domains or interfaces between them and thereby generate synergy in their modes of correction. The analysis of the current combination compounds has been described in the studies of Veit et al. (2020) and Laselva et al. (2021a). The location of the CFTR2 mutants in different CFTR domains, together with the concept that the domains of CFTR fold cooperatively, suggests that these cooperative interactions require different pharmaco-chaperones to optimize their overall folding efficacy. It has also been suggested that these CFTR domain interactions occur posttranslationally (Du and Lukacs, 2009; Kleizen et al., 2021) and are coupled to one another, consistent with the concept that folding defects arising from missense mutations in different CFTR domains would be distinct and require compounds that target different steps in the cooperative folding pathway of CFTR.

Intransigent mutants included missense mutations such as G85E, V520F, R560K, R560T, and R1066C. These variants produced no measurable band C with the combination of corrector C18 plus PIAS4. Thus, despite the increase in immature CFTR produced by PIAS4, the efficacy of corrector C18 under these conditions was insignificant.

Prior studies of G85E and V520F have illustrated the relative intransigence of these mutations to respond to single correctors that resemble C18. Human nasal epithelial (HNE) cells homozygous for G85E/G85E did not respond to VX-661 but responded to the second-generation correctors, i.e., VX-445 are currently available to patients with CF in a form that also includes the CFTR potentiator, VX-770; this threesome is termed as “TRIKAFTA^TM^.” Similar findings have been described in the studies of the R560K and R560T CFTR variants, and in a recent study, these mutants were not sensitive to recently developed correctors. In addition, V520F HNE was examined in primary epithelia that also had the splice mutation, 1717-1G- > A, on the second allele; this splice mutation genotype is not considered to give rise to significant protein expression (Sosnay et al., 2013) so that the combined genotype would reveal the properties of the V520F allele. Based on the prior functional studies, it is possible that a small level of CFTR folding correction of these intransigent mutants could be obtained by combining a type I corrector (e.g., VX-661) with the type III corrector, VX-445, although the corrector efficacy for this group remains relatively low (Veit et al., 2020; Laselva et al., 2021b), on the order of 5% of WT CFTR. This outcome is generally considered to be of limited clinical value.

Throughput responsive mutants included the common mutant, F508del, and the missense mutations such as E92K, R347P, A455E, S492F, and M1101K. These variants generated some band C in response to C18, but quantitatively, the relative amount of mature protein produced was less than that expected from the increase in band B associated with the action of PIAS4 on immature CFTR expression. The slope of the line describing this dataset was 0.96, i.e., there was no obvious amplification of the corrector response, which should have generated a slope greater than 1.0.

The canonical F508del mutation falls into this group; it is present in ∼90% of patients with CF on at least one allele. The folding pathway(s) of CFTR have been examined in detail for F508del due to its genomic prevalence. These studies have shown that the folding of CFTR F508del is managed by a set of cytosolic components that dictate the ability of the protein to traverse the ERQC pathways that determine the opportunity for CFTR transport from the ER through the Golgi complex to the apical PM, as well as the stability of CFTR at the PM. As noted earlier, F508del is frequently in combination with the second allele in heterozygous CFTR genotypes, which complicates treatment of difficult to correct CFTR2 alleles whose properties are poorly understood.

Another variant associated with the throughput responsive group was R347P; it is a somewhat common mutation in the Netherlands, where it comprises approximately 4% of CF cases, usually expressed as a single allele. In terms of a mechanistic classification, this mutation was assigned originally as a class IV gating mutant, analogous to G551D. However, subsequent study, and the interim development of more effective CFTR modulators, permitted its reclassification as a class II mutant with a serious misfolding phenotype (van Willigen et al., 2019). The analysis of its response to VX-770 did not improve its function as would have been expected for a class IV mutant; however, incubation with VX-809 corrected its misfolding, and the properties identified in several class IV mutants revealed that R347P had been misassigned. The mutation, in this case, lies in TM6, where, as predicted from the cryo-EM structure of CFTR (Liu et al., 2017; Zhang et al., 2017; van Willigen et al., 2019), the WT arginine at position 347 forms a salt bridge to an aspartate that allows it to pack closely to ICL1 and the N-terminus of MSD1. The corrector VX-809 is considered to improve the packing of MSD1 and to rescue its interactions with NBD1, providing this corrector with broad-ranging activity, including the rescue of R347P CFTR. Relative to WT CFTR, only ∼15% of R347P matures and traffics to the PM, consistent with its misfolding molecular phenotype.

Another throughput responsive mutant in our study group was A455E. The examination of four rare mutations, namely, E60K, G85E, E92K, and A455E, each of which comprise <1% of the mutant population in the CFTR2 database, has produced findings similar to those presented in this study (Ensinck et al., 2020). In the previous study, the Western blot of A455E produced some band C, which was marginally detectable, even after the immature protein was increased through exogenous expression, which is similar to our experience with PIAS4 and the A455E variant. Its ability to mature was similar to that of F508del CFTR, the founding member of the throughput responsive group. In contrast, VX-809 produced significant maturation responses from E60K and E92K, but the increased band C production from A455E was significantly lower than observed from those variants. The same was true of the functional responses of these mutants in forskolin-induced swelling assays performed on intestinal organoids (Ensinck et al., 2020).

Folding responsive mutants included P67L, R117H, R334W, S549R, D614G, L1065P, L1077P, and N1303K. These mutants generated more band C in response to corrector C18 than that expected from the relative increase in immature CFTR associated with the expression of PIAS4; that is, PIAS4 amplified the corrector response from these mutants.

Among these mutants, P67L has been categorized as a mutation that interferes with anion transport based on protein misfolding and reduced maturation (Sabusap et al., 2016). This mutant also shows channel gating defects, but it is thermally stable and exhibits normal conductance properties. Importantly, P67L CFTR expression was diminished relative to that of WT CFTR, but it was markedly augmented by VX-661 when expressed in Fischer rat thyroid (FRT) cells due to increased protein stability. The corrector also significantly augmented its anion secretion activity. In addition, improved clinical outcomes were noted in a patient with the P67L variant on one allele. These findings, suggestive of relatively mild disease, have been supported by other observations (Yousef et al., 2015) including the presence of pancreatic sufficiency in patients with this genotype (Gilfillan et al., 1998; Supplementary Table 1). Accordingly, P67L displays the characteristics and responses to modulators that are expected of a folding responsive mutant.

An R117H exhibited a phenotype that was similar to P67L and several other members of the folding responsive group. Heterozygous with F508del, this genotype has been studied extensively. The site of this mutation lies in the extracellular loop that connects the first and second transmembrane helices of MSD1 (Loo and Clarke, 2017). The mutation is often combined with a 7T polymorphism in the poly-T tract of exon 8, and this has been associated with less severe cases of CF disease as opposed to the 3T and 5T repeats, which are more prevalent but less responsive (de Nooijer et al., 2011; van Mourik et al., 2020). Clinically, patients with R117H have been treated with VX-770 (Ivacaftor), but functional responses observed in the cells of the patient suggested that corrector treatment can also be useful. A patient with an R117H-7T/F508del genotype was studied by Park et al. (2020) using the Epix technology to expand nasal cells and to provide for functional studies. The cells exhibited robust responses to forskolin and VX-770 that were blocked by CFTR inhibitors. The forskolin-stimulated current amounted to 40% of the WT control current, even without treatment with a potentiator or a corrector. More significant secretory responses were evoked by treatment of nasal cultures with correctors VX-809 and VX-661. These data agree with the designation of a mild phenotype with intermediate CFTR function. The responses of the R117H/F508 nasal cells were in a similar range as those reported by Gentzsch et al. (2016), and they confirmed a relatively mild phenotype.

Finally, another mutant whose category designation should be discussed is N1303K. This NBD2 mutation has been difficult to correct using early compounds such as VX-809, and it is similarly unphased by VX-661; both correctors act to stabilize NBD1 and its association with intracellular loops emerging from the membrane domains. Studies of N1303K using more recent compounds and the triple combination, i.e., VX-445 + VX-661 + VX-770, showed significant rescue of missense mutations such as N1303K and others that are found in MSD1, MSD2, NBD1, and NBD2 of CFTR by Veit et al. (2020), who also suggested that the findings represent a concerted correction mechanism in which TRIFAKTA^TM^ would bring relief to patients with rare misfolding mutants of CFTR. This still begs the question of why N1303K appears to be more difficult to correct in some systems, even in some studies of patient-derived cells studied *in vitro*. A recent study by He et al. (2021) suggests that this may represent the presence of a different pathway involving ER-selective autophagy that diverts some misfolded proteins from endoplasmic reticulum associated degradation (ERAD), i.e., the proteasome-targeted pathway that degrades most CFTR missense mutants, and that N1303K is excluded from ER exit sites and instead passes to autolysosomes that triage selected protein intermediates. Whether this additional pathway for the degradation of CFTR misfolding mutants varies among cell types or experimental conditions is a question for future study.

## Conclusion

The data presented in this study, and the selective behavior of different folding mutants when immature CFTR expression was amplified by PIAS4, are consistent with structure-dependent steps in the cooperative folding landscape of CFTR that can be selectively modulated by pharmaco-chaperones. Thus, it would be of interest to use this amplification approach to test available and emerging corrector compounds to determine whether consistent or different patterns of behavior are observed when challenged with compounds other than C18/VX-809. Investigation into these pathways may permit the determination of whether the PIAS4-associated SUMOylation pathway, which enhances early steps in CFTR biogenesis, can be modulated with small molecules to enhance the efficacy of correctors of mutant CFTR folding (Ahner and Frizzell, 2015). In this respect, our principal focus would be on variants in the less-approachable intransigent and throughput responsive categories.

## Data Availability Statement

The raw data supporting the conclusions of this article will be made available by the authors, without undue reservation.

## Author Contributions

RF and KP conceived of the project, organized the manuscript and the figures, contributed to the writing, reviewed drafts, and approved the final version of the manuscript. XG performed the cycloheximide chase experiments and tabulated the data. KP performed the remainder of the experiments and tabulated and analyzed their data. All authors contributed to the article and approved the submitted version.

## Conflict of Interest

The authors declare that the research was conducted in the absence of any commercial or financial relationships that could be construed as a potential conflict of interest.

## Publisher’s Note

All claims expressed in this article are solely those of the authors and do not necessarily represent those of their affiliated organizations, or those of the publisher, the editors and the reviewers. Any product that may be evaluated in this article, or claim that may be made by its manufacturer, is not guaranteed or endorsed by the publisher.

## Footnotes

1
www.cftr2.org

2
https://clinicaltrials.gov/ct2/show/NCT00865904
