# Supplementary material for: Cystic Fibrosis Transmembrane Conductance Regulator Folding Mutations Reveal Differences in Corrector Efficacy Linked to Increases in Immature Cystic Fibrosis Transmembrane Conductance Regulator Expression
Source: Front Physiol. 2021 Oct 26;12:695767. doi: 10.3389/fphys.2021.695767 (PMC8576290; doi:10.3389/fphys.2021.695767)
Supplement: Supplementary file 1 [file Table_1.pdf]

**Table 1.S: Analysis of CFTR Missense Mutations**

| | CFTR2 variant annotation (as of 31 July 2020) | n alleles in CFTR2 | Sweat chloride, mEq/L[a] | % Patients who are PI[a,b] |
|---|---|---|---|---|
| Non-CF | - | - | Less than 30 | Less than 1% |
| P67L | CF-causing | 239 | 60 | 33 |
| G85E | CF-causing | 610 | 100 | 86 |
| E92K | CF-causing | 38 | 89 | 54 |
| R117H;9T[c] | - | - | - | - |
| R117H;7T | Varying clinical consequences | 125 | 43 | 15 |
| R117H;5T | CF-causing | 102 | 74 | 31 |
| R334W | CF-causing | 425 | 99 | 40 |
| R347P | CF-causing | 528 | 100 | 67 |
| A455E | CF-causing | 496 | 84 | 34 |
| S492F | CF-causing | 24 | 79 | 48 |
| V520F | CF-causing | 156 | 105 | 96 |
| R560K | CF-causing | 15 | 110 | 100 |
| R560T | CF-causing | 343 | 103 | 98 |
| S549R | CF-causing | 93 | 105 | 98 |
| D614G | Varying clinical consequences | 17 | 60 | 29 |
| L1065P | CF-causing | 43 | 103 | 79 |
| R1066C | CF-causing | 220 | 104 | 98 |
| L1077P | CF-causing | 96 | 101 | 94 |
| M1101K | CF-causing | 238 | 106 | 70 |
| N1303K | CF-causing | 2226 | 105 | 98 |

[a]Clinical data shown from individuals in CFTR2 who have variant of interest and a PI-CF-causing variant

[b]PI; pancreatic insufficient

[c]Not annotated on cftr2.org
